# The Kynurenine 3-Monooxygenase Encoding Gene, *BcKMO*, Is Involved in the Growth, Development, and Pathogenicity of *Botrytis cinerea*

**DOI:** 10.3389/fmicb.2018.01039

**Published:** 2018-05-18

**Authors:** Kang Zhang, Xuemei Yuan, Jinping Zang, Min Wang, Fuxin Zhao, Peifen Li, Hongzhe Cao, Jianmin Han, Jihong Xing, Jingao Dong

**Affiliations:** ^1^Key Laboratory of Hebei Province for Plant Physiology and Molecular Pathology, Hebei Agricultural University, Baoding, China; ^2^Mycotoxin and Molecular Plant Pathology Laboratory, Hebei Agricultural University, Baoding, China

**Keywords:** *Botrytis cinerea*, *BcKMO*, kynurenine 3-monooxygenase, growth, development, pathogenicity

## Abstract

A pathogenic mutant, BCG183, was obtained by screening the T-DNA insertion library of *Botrytis cinerea*. A novel pathogenicity-related gene *BcKMO*, which encodes kynurenine 3-monooxygenase (KMO), was isolated and identified via thermal asymmetric interlaced PCR, bioinformatics analyses, and KMO activity measurement. The mutant BCG183 grew slowly, did not produce conidia and sclerotia, had slender hyphae, and presented enhanced pathogenicity. The phenotype and pathogenicity of the *BcKMO*-complementing mutant (BCG183/*BcKMO*) were similar to those of the wild-type (WT) strain. The activities of polymethylgalacturonase, polygalacturonase, and toxins were significantly higher, whereas acid production was significantly decreased in the mutant BCG183, when compared with those in the WT and BCG183/*BcKMO*. Moreover, the sensitivity of mutant BCG183 to NaCl and KCl was remarkably increased, whereas that to fluconazole, Congo Red, menadione, H_2_O_2_, and SQ22536 and U0126 [cAMP-dependent protein kinase (cAMP) and mitogen-activated protein kinase (MAPK) signaling pathways inhibitors, respectively] were significantly decreased compared with the other strains. Furthermore, the key genes involved in the cAMP and MAPK signaling pathways, *Pka1*, *Pka2*, *PkaR*, *Bcg2*, *Bcg3*, *bmp1*, and *bmp3,* were significantly upregulated or downregulated in the mutant BCG183. *BcKMO* expression levels were also upregulated or downregulated in the RNAi mutants of the key genes involved in the cAMP and MAPK signaling pathways. These findings indicated that *BcKMO* positively regulates growth and development, but negatively regulates pathogenicity of *B. cinerea*. Furthermore, *BcKMO* was found to be involved in controlling cell wall degrading enzymes activity, toxins activity, acid production, and cell wall integrity, and participate in cAMP and MAPK signaling pathways of *B. cinerea*.

## Introduction

*Botrytis cinerea*, a typical necrotrophic pathogenic fungus, infects more than 200 plant species worldwide, causing significant economic losses to agricultural production (Elad et al., 2007; Williamson et al., 2007). It is characterized by abundant conidia and sclerotia, and can overwinter through intact mycelia or sclerotia, which germinate in spring to produce conidia. The conidia are dispersed by wind and rainwater, and cause the next cycle of infections. Identification of the functional genes regulating the growth, development, and pathogenicity of *B. cinerea* is very important to understand the mechanism of this fungal dispersion. With the completion of genome sequencing of *B. cinerea* strains B05.10 and T4, *B. cinerea* has become a model organism in the field of developmental biology and molecular plant pathology.

*Botrytis cinerea* produces various virulence factors to penetrate the host surface, kill the host cells, and generate lesions (Choquer et al., 2007). Among them, multiple cell wall degrading enzymes (CWDEs) and non-specific pathotoxins secreted by *B. cinerea* are considered to be the major pathogenic factors. For instance, pectinase secreted by *B. cinerea* causes leaf rot, leading to the collapse of chloroplasts and mitochondrion of leaf cells. Several types of CWDEs are extracellularly secreted by *B. cinerea* at the stages of conidial germination and hyphal growth (Blanco-Ulate et al., 2014), among which polymethylgalacturonase (PMG) exhibits superior activity (Van Kan, 2006). Furthermore, it has been reported that the polygalacturonase (PG), BcPG1, contributes to penetration and is essential during colonization of *B. cinerea* (ten Have et al., 1998), whereas BcPG2 contributes only to penetration (Choquer et al., 2007; Patel et al., 2008). Moreover, inactivation of the pectin methylesterase (PME) gene, *bcpme1*, in *B. cinerea* has been found to result in a sharp reduction in the fungal virulence on several plant hosts, suggesting that PMEs might facilitate the action of PG (Valette-Collet et al., 2003). Cellulase (CX), hemicellulases and non-pectinolytic CWDEs also promote *B. cinerea* infection (Choquer et al., 2007). *B. cinerea* secretes nearly 20 types of non-specific pathotoxins, including polyketide botcinic acid and sesquiterpene botrydial, to support both penetration and colonization. Botrydial was first isolated and described in 1974, and has been reported to exhibit highest phytotoxic activity (Williamson et al., 2007). The genes involved in the botrydial biosynthetic pathway have been identified as a physical cluster, including *BcBOT1*, *BcBOT2*, *BcBOT3*, *BcBOT4*, and *BcBOT5*, among which *BcBOT2* encodes sesquiterpene synthase that is responsible for the committed step in botrydial biosynthetic pathway (Pinedo et al., 2008). Sesquiterpene synthase converts farnesyl diphosphate to the precursor of botrydial, presilphiperfolan-8β-ol (PSP; the tricyclic sesquiterpene alcohol). PSP is converted to botrydial by the action of three cytochrome P450s (encoded by *BcBOT1*, *BcBOT3*, and *BcBOT4*) and an acetyl transferase (encoded by *BcBOT5*) (Wang et al., 2009). It has been reported that deletion of *BcBOT2* eliminated the production of botrydial and resulted in strain-dependent loss of virulence (Pinedo et al., 2008).

In addition to CWDEs and pathotoxins, *B. cinerea* produces reactive oxygen species and oxalic acid during infection (Van Kan, 2006), and H_2_O_2_ accumulation has been noted in germinating spores, infection cushions, and in the early stages of *B. cinerea* infection. Deletion mutants of *B. cinerea* superoxide dismutase (*BcSOD1*; contributing to the accumulation of phytotoxic H_2_O_2_ levels) have been found to exhibit significantly retarded development of lesions, indicating that the H_2_O_2_-generating enzyme in *B. cinerea* is a virulence factor (Rolke et al., 2004). Furthermore, *B. cinerea* could produce non-host specific toxin, oxalic acid (OA), both *in vivo* and *in vitro*, which is often described as an important factor in the pathogenesis of *Sclerotinia sclerotiorum* (Walz et al., 2008). However, several reports have suggested that OA is a less important factor in *B. cinerea* infection, and that *B. cinerea* exploits the plant defense mechanisms for its growth and proliferation in the plant tissue.

Several genes of signal transduction pathways, such as the mitogen-activated protein kinase (MAPK), cAMP-dependent protein kinase (cAMP), and Ca^2+^/calcineurin pathways, have been characterized, and their effects on the growth, development, and pathogenicity of *B. cinerea* have been reported (Doehlemann et al., 2006; Choquer et al., 2007). The Slt2-type MAPK *Bmp3* mutant has been reported to show significantly impaired conidiation, reduced vegetative growth, and loss of sclerotium formation (Rui and Hahn, 2007; Schamber et al., 2010), while the mutant of *bos5* encoding a MAPK kinase has been found to exhibit severely impaired conidiation, reduced vegetative growth, and loss of pathogenicity (Yan et al., 2010). Furthermore, deletion mutants of the MAPK encoding gene, *bcsak1*, have been observed to present significantly impaired growth, conidia formation, sclerotia development, and pathogenicity (Segmuller et al., 2007; Heller et al., 2012), while in the BcPKA1 mutant, the catalytic subunit of the cAMP protein kinase A (PKA), demonstrated impaired growth, colony morphology, and virulence (Schumacher et al., 2008b). Moreover, deletion of BcCRZ1, a calcineurin-responsive zinc finger transcription factor, has been found to cause severely impaired growth, hyphal morphology, sclerotia and conidia formation, and pathogenicity (Schumacher et al., 2008a), and, so far, more than 100 genes involved in the growth, development, and pathogenicity of *B. cinerea* have been reported (Choquer et al., 2007).

Kynurenine 3-monooxygenase (KMO) is a NADPH-dependant flavoprotein hydroxylase and an important enzyme located at an important branch of the kynurenine pathway (Smith et al., 2016). Kynurenine pathway is the primary route of synthesis of the essential cellular cofactor NAD, and is responsible for >95% of tryptophan metabolism in mammals (Wilson et al., 2014). KMO belongs to the family of oxidoreductases, catalyzes kynurenine to generate 3-hydroxykynurenine in the kynurenine pathway. Currently, KMO is an attractive drug target for several immunological, neurodegenerative, and neuroinflammatory diseases, especially Alzheimer’s, Huntington’s, and Parkinson’s disease (Smith et al., 2016). The kynurenine pathway has long been considered to be specific for eukaryotic organisms only, but later, several genes of the kynurenine pathway were identified in several bacteria and shown to be functional (Kurnasov et al., 2003a,b). The kynurenine pathway was involved in the biosynthesis of a quinoline siderophore, actinomycin, and benzodiazepine in *Pseudomonas siderophore*, *Streptomyces chrysomallus*, and *S. refuineus*, respectively (Matthijs et al., 2004; Hu et al., 2007; Keller et al., 2010). So far, there is no report on the presence of kynurenine pathway and KMO in *B. cinerea*.

In the present study, a novel pathogenic mutant, BCG183, screened from the T-DNA insertion library of *B. cinerea* was described, and a newly identified locus, *BcKMO* (BC1G_07455), which encodes a hypothetical protein similar to kynurenine 3-monooxygenase (KMO), was examined. Furthermore, the CWDEs activity, toxins activity, and expression levels of pathogenicity-related genes were investigated in the *B. cinerea* wild-type (WT), *BcKMO* T-DNA insertion mutant (BCG183), and *BcKMO*-complementing mutant (BCG183/*BcKMO*). The findings of this study could contribute to better understanding of the regulatory mechanisms of *BcKMO* in the growth, development, and pathogenicity of *B. cinerea*.

## Materials and Methods

### Strains and Culture Conditions

The WT *B. cinerea* strain BC22 was isolated by the Mycotoxin and Molecular Plant Pathology Laboratory, Agriculture University of Hebei, China. The mutant BCG183 was obtained from the ATMT mutant library of the *B. cinerea* WT strain BC22. All the *B. cinerea* strains were cultured on potato dextrose agar (PDA) at 22°C. For DNA and RNA extraction, the mycelia were grown in potato dextrose (PD) medium for 5–7 days at 22°C. For protoplasts preparation, the mycelia from the mutant BCG183 were cultivated in malt/yeast medium for 24 h with shaking.

### Identification of Mutant BCG183

Genomic DNA was isolated from the WT and the BCG183 mutant by cetyltrimethylammonium bromide method, and used as a PCR template along with the specific primers of hygromycin B phosphotransferase gene (*hph*). Southern blot was performed to confirm the presence of T-DNA in the genomic DNA of the mutant BCG183 by using specific fragment of the *hph* gene as the probe. The precise fragment of the *hph* gene was amplified with *hph* gene specific primers, purified, and labeled with digoxigenin (DIG) using a DIG Labeling Kit (Roche, Germany). The genomic DNA of the mutant BCG183 was isolated and digested by *Hind*III, electrophoretically separated on 0.8% (w/v) agarose gel, and transferred to a nylon membrane (Sangon Co., Ltd., China). Hybridization was performed for 16 h at 48°C in PerfectHyb plus hybridization buffer (Sigma-Aldrich, United States), and immunological detection was conducted overnight.

### Identification of Mutant Gene in BCG183

The flanking sequence of the T-DNA insert was obtained by thermal asymmetric interlaced PCR (TAIL-PCR), as described previously (Mullins et al., 2001). The genomic DNA of the mutant BCG183 was used as a template along with AD4 primer and LB1/LB2/LB3 primer (**Supplementary Table S1**). The tertiary PCR products were electrophoresed on a 1.0% TAE-agarose gel and purified with QIAquick columns (TIANgel Midi Purification Kit, Cat. No. Dp209-03, Qiagen, Germany). The purified PCR products were cloned into the plasmid pMD-18 vector (Takara, Japan) and sequenced by Sangon Co., Ltd., China. The T-DNA insert locus was obtained using BLAST program with *B. cinerea* genome sequences in the *B. cinerea* genome database^1^.

Semi-quantitative RT-PCR was used to determine the expression levels of the T-DNA insert gene and confirm the presence of the mutant gene in the mutant BCG183. The total RNA was isolated from the WT and mutant BCG183 with a Total RNA Preparation Kit (Omega Bio-Tek, Inc., United States), according to the manufacturer’s instructions. Reverse transcription was performed with RevertAid M-MuLV Reverse Transcriptase (Sangon Co., Ltd., China). The synthesized cDNA served as the PCR template, whereas *tubulin* gene was used for equal loading.

### Bioinformatics Analysis of the Mutant Gene *BcKMO*

The conserved domain of the BcKMO protein was obtained using the BLAST-based NCBI conserved domain search engine (Marchler-Bauer et al., 2017). Amino acid sequence homology alignment of the BcKMO protein was performed using BLAST programs of NCBI^2^. Phylogenetic analysis of BcKMO and related proteins was performed using MEGA7 software (Kumar et al., 2016).

### KMO Activity Measurements

The purified BcKMO protein was obtained by prokaryotic expression analysis of *BcKMO* from *B. cinerea,* and the KMO activity was measured using Human KMO ELISA Kit (DLDEVELOP, Canada) based on sandwich enzyme immunoassay. The enzyme–substrate reaction was terminated by the addition of sulfuric acid solution, and the color change was measured spectrophotometrically at a wavelength of 450 ± 10 nm. The concentration of KMO in the samples was determined by comparing the optical density (OD) values of the samples to the standard curve.

### Complementation of the *BcKMO* Gene

The *BcKMO*-coding region was amplified with *BcKMO*-specific primers and cloned into pBARKS1-eGFP using *Sac*I and *Sma*I restriction sites, to obtain pBARKS1-*BcKMO*-eGFP (**Supplementary Figure S3**). The protoplasts of the mutant BCG183 were prepared and transformed with pBARKS1-*BcKMO*-eGFP as described previously (Levis et al., 1997). PCR of the transformant with *GFP-* and *bar*-specific primers showed that the constructs were integrated. Southern blot was used to confirm the integration of the construct with *GFP*-specific probe. Quantitative real-time PCR experiments established that the introduced construct was expressed in the transformant.

### Phenotypic Analyses

For phenotypic analyses of the mutant BCG183 and complement mutant, the mycelia of the WT, mutant BCG183, and *BcKMO*-complementing mutant were, respectively, inoculated onto PDA plates and incubated for 10 days at 22°C. The color, morphology, and sclerotia formation of the colonies were observed. The morphology and septum spacing of the hyphae were determined under a microscope (Nikon Eclipse E-200). For conidiation assays, 10-day-old conidia of all the strains were collected with 7 mL of sterile water per PDA plate and counted under a microscope. For growth assays, 5 mm of mycelial blocks of all the strains were incubated at the center of PDA plates, and the colony diameters were measured every 24 h for 7 days. All experiments were performed in triplicate.

### Pathogenicity Assay

Detached kidney beans and cucumber leaves were used for the pathogenicity assay. The leaves were washed 2–3 times with sterile water, sterilized with 75% alcohol for 3 min, and washed again with sterile water. Then, culture blocks of the WT and mutants were prepared and inoculated onto the intact leaves. The inoculated leaves were moisturized and incubated at 22°C, and observed daily. Lesion formation was examined at 4 days post-inoculation (dpi). The assay was performed in triplicate.

### CWDEs Activity Assays

Cell wall degrading enzymes from *B. cinerea* were extracted and assayed based on a previously described method (Berto et al., 2001). The WT and mutants were, respectively, cultured for 10 days in liquid pectin and cellulose media at 22°C. Subsequently, the cultured media were filtered and centrifuged (8500 r/min) to remove the fungal mycelia, and the CWDEs were extracted and purified from the filtrates. PMG, PG, and CX activities were analyzed by the DNS method. The Hoffman method was used to determine the activities of polygalacturonic acid transeliminase (PGTE) and pectin methyl transelimination enzyme (PMTE). All the experiments were repeated thrice.

### Toxins Activity Assay

The WT and mutants were, respectively, inoculated into Fries III medium, and cultured for 21 days at 22°C. Then, the culture filtrates of each strain were extracted thrice with ethyl acetate, and evaporated under reduced pressure to remove the solvent. The final extracts of each strain were dissolved in chromatographic methanol and inoculated into punctured tobacco leaves. The inoculated leaves were moisturized in a cylinder culture dish at 22°C and observed daily. The lesion areas were measured at 4 dpi.

### Acid Production Assays

The WT and mutants were, respectively, inoculated onto PDA media supplemented with 0.05% (w/v) bromothymol blue, a pH indicator for weak acids and bases, and incubated in complete darkness at 22°C for 7 days. A change in the color of the media to yellow suggested that the pH of the media declined and the strains secreted acids. For pH detection, the WT and mutants were, respectively, inoculated in 100 mL of PD media and cultured for 7 days in darkness at 22°C. All the experiments were performed at least thrice.

### Assessment of Mutant Cell Wall Integrity

To assess the mutant cell wall integrity, the WT and mutants were inoculated onto PDA media supplemented with NaCl, KCl, fluconazole, Congo Red, menadione, and H_2_O_2_ at optimal concentrations of 0.8 mol/L, 0.8 mol/L, 10 μg/mL, 2 mg/mL, 200 μmol/L, and 30 mmol/L, respectively, and were incubated in complete darkness at 22°C. The growth and inhibition rates of the WT and mutants were determined.

### H_2_O_2_ Measurements

All samples from WT, BCG183 and BCG183/*BcKMO* mutant were homogenized in liquid nitrogen. Using the H_2_O_2_ Quantitative Assay Kit (Sangon Co., Ltd., China), we followed the manufacturer’s instructions to measure the quantity of H_2_O_2_ production in WT, BCG183 and BCG183/*BcKMO*.

### Sensitivity of Mutants to Inhibitors of cAMP and MAPK Signaling Pathways

SQ22536 and U0126, the specific inhibitors of cAMP and MAPK signaling pathways, respectively, were used to detect the sensitivity of the WT and mutants to the inhibitors of cAMP and MAPK signaling pathways. The WT, BCG183, and BCG183/BcKMO were inoculated onto PDA media supplemented with 10 μmol/L SQ22536 or 10 μmol/L U0126, respectively, and the growth and inhibition rates of the strains were determined.

### Analysis of cAMP Content in the Mutants

The cAMP in the WT, BCG183 and BCG183/*BcKMO* was extracted by bath method and detected by using Agilent 1100 series high-performance liquid chromatography (HPLC) system (Agilent, United States). HPLC was performed with Kromasil C18 anti-phase chromatographic column (250 mm × 4.6 mm, 5 μm) under the following conditions: flow rate, 0.8 mL/min; V (methanol):V (KH_2_PO_4_), 80:20; testing wave, 254 nm; column temperature, 30°C; and sample injection volume, 10 μL.

### Construction of RNAi Mutants of Key Genes Involved in the cAMP and MAPK Signaling Pathways

The explicit fragments of the key genes involved in the cAMP and MAPK signaling pathways, including *pka1*, *pka2*, *pkaR*, *bcg2*, *bcg3*, *bmp1*, and *bmp3*, were amplified with specific primers using WT cDNA as the template, and linked into pCR8 vector to obtain the entry vectors. The appropriate entry vectors of the key genes involved in cAMP and MAPK signaling pathways were obtained by colony PCR screening and sequencing. The RNAi vectors of *pka1*, *pka2*, *pkaR*, *bcg2*, *bcg3*, *bmp1*, and *bmp3* were obtained by LR reaction between the entry vectors and destination vector pK7GW1WG2. Then, by using the Agrobacterium-mediated method, the RNAi vectors of the key genes involved in the cAMP and MAPK signaling pathways were transferred into the WT, respectively. The RNAi mutants of *pka1*, *pka2*, *pkaR*, *bcg2*, *bcg3*, *bmp1*, and *bmp3* were obtained through PCR, and real-time PCR identification with primers specific for the *kan* gene revealed that the RNAi vectors were integrated. Furthermore, real-time PCR with primers specific for *pka1*, *pka2*, *pkaR*, *bcg2*, *bcg3*, *bmp1*, and *bmp3* confirmed that the expression levels of these genes were affected.

### Quantitative Real-Time PCR Analysis

Quantitative real-time PCR was used to measure the transcript levels of *BcKMO* and key genes of the cAMP and MAPK signaling pathways, such as the PKA catalytic subunit genes, *pka1* and *pka2* (Schumacher et al., 2008b); the PKA regulatory subunit gene, *pkaR* (Schumacher et al., 2008b); subunits of heterotrimeric GTP binding protein encoding genes, *bcg2* and *bcg3* (Gronover et al., 2001); and MAPK genes including, *Bmp1* and *Bmp3* (Segmuller et al., 2007; Heller et al., 2012), using ABI SYBR Green PCR Master Mix (Applied Biosystems, United States). The total RNA of each strain was isolated by Total RNA Preparation Kit (Omega), and digested with DNase I (Promega) to remove genomic DNAs. The cDNAs were synthesized with a Revert Aid First-strand cDNA Synthesis Kit (Invitrogen), according to the manufacturer’s instructions; the *tubulin* gene was used as an internal standard for equal loading.

## Results

### *BcKMO* Encodes a KMO

**FIGURE 1:**
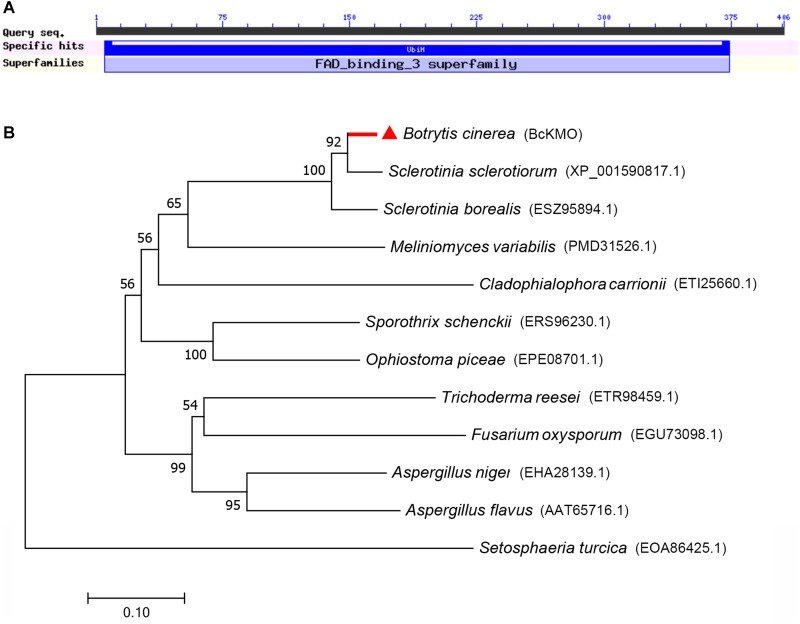
Bioinformatics analysis of BcKMO. **(A)** Conserved domains of BcKMO. The domain of BcKMO was predicted using NCBI’s conserved domain database. **(B)** Phylogenetic analysis of BcKMO and homologous proteins from other fungi. The full-length protein sequences were analyzed by MEGA 7.0 with unrooted neighbor-joining bootstrap (1000 replicates).

A pathogenic mutant, BCG183, was isolated by screening the T-DNA insertion library of *B. cinerea*. The mutant gene of BCG183 was identified as BC1G_07455 by TAIL-PCR and semi-quantitative RT-PCR (**Supplementary Figure S1**). Sequence searches in the databases revealed that BC1G_07455 encodes a hypothetical protein similar to KMO with a monooxygenase flavin-adenine dinucleotide (FAD) binding domain and four aromatic-ring hydroxylase-like motifs (**Figure 1A**), indicating that BcKMO might have similar functions to its ortholog in animals. A phylogenetic tree was constructed to update the new functional clades using MEGA software (**Figure 1B**). BcKMO displayed high homology with the Rossmann-fold NAD(P)(+)-binding proteins of *S. sclerotiorum* and ubiquinone biosynthesis hydroxylase family proteins of *S. borealis*, which contain a conserved FAD-binding domain.

The purified BcKMO protein was obtained using a prokaryotic expression technique (**Supplementary Figure S2**), and its KMO activity was measured using the Human KMO ELISA Kit. Based on the concentration and OD of the standard, the standard curve of KMO activity was derived and the linear regression equation of the standard curve was calculated as *y* = 0.0171x + 0.0623 (**Supplementary Figure S2E**). The activity of the purified BcKMO protein was 2.56 U/L. These results suggested that *BcKMO* encodes a KMO in *B. cinerea*.

### *BcKMO* Is Important for Growth and Development of *B. cinerea*

**FIGURE 2:**
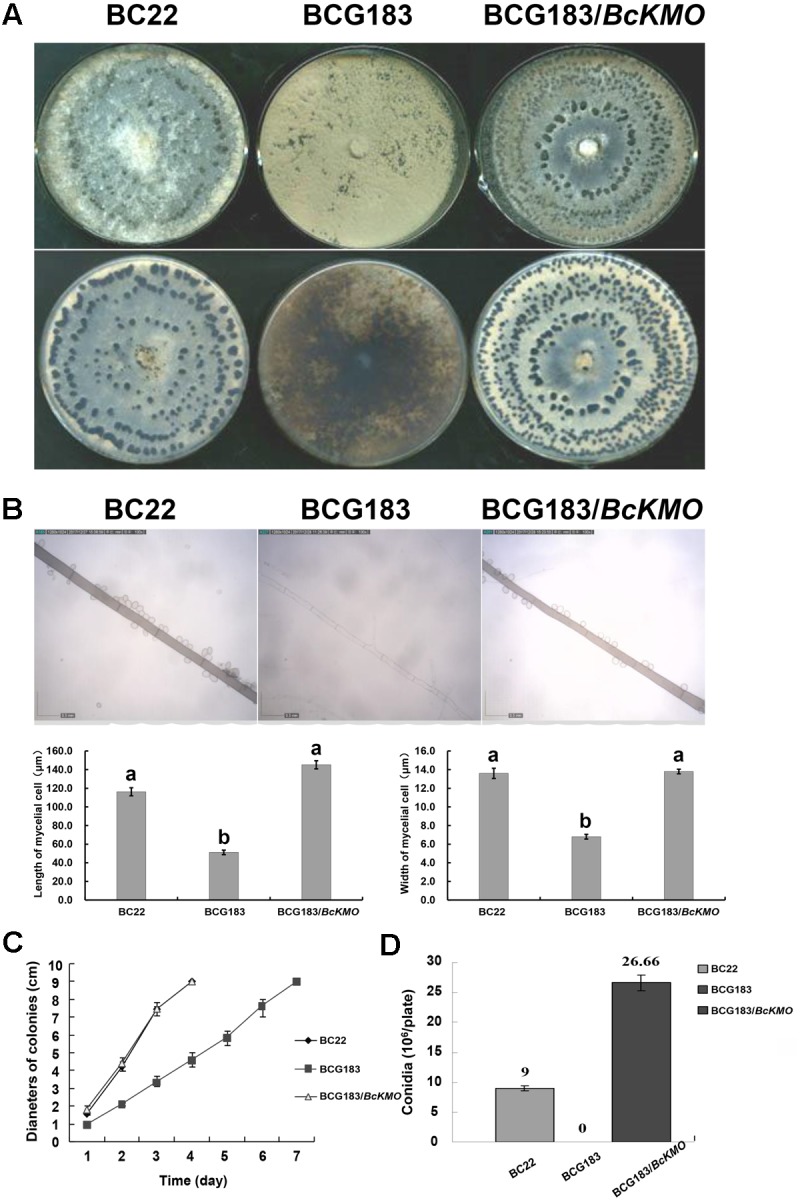
Phenotype analysis of BCG183 and BCG183/*BcKMO* mutants. **(A)** Colony morphology (10 dpi). **(B)** Mycelium morphology; CE: BcKMO-complementing BCG183/*BcKMO* mutant. Data in the table were averaged to evaluate statistical significances among the strains; a and b indicate significant differences at 0.05 probability level. **(C)** Detection of growth rates. **(D)** Determination of conidial yield.

To determine the role of *BcKMO* in the growth and development of *B. cinerea*, colony morphology, mycelium morphology, growth rate, and conidial yield of the WT, BCG183, and BCG183/*BcKMO* strains were investigated. The WT and BCG183/*BcKMO* colonies were taupe-colored and produced large amounts of sclerotia. In contrast, the BCG183 colonies were gray and did not produce sclerotia (**Figure 2A**). Under optical microscope, the BCG183 mycelia were white and slender with shorter transverse septa, when compared with the WT and BCG183/*BcKMO* mycelia (**Figure 2B**). Furthermore, the growth rate of the mutant BCG183 was significantly lower than that of the WT and BCG183/*BcKMO* (**Figure 2C**). Conidiation assays showed that the BCG183 mutant did not produce conidia, whereas the WT and BCG183/*BcKMO* strains did (**Figure 2D**).

### *BcKMO* Is Vital for *B. cinerea* Pathogenicity

**FIGURE 3:**
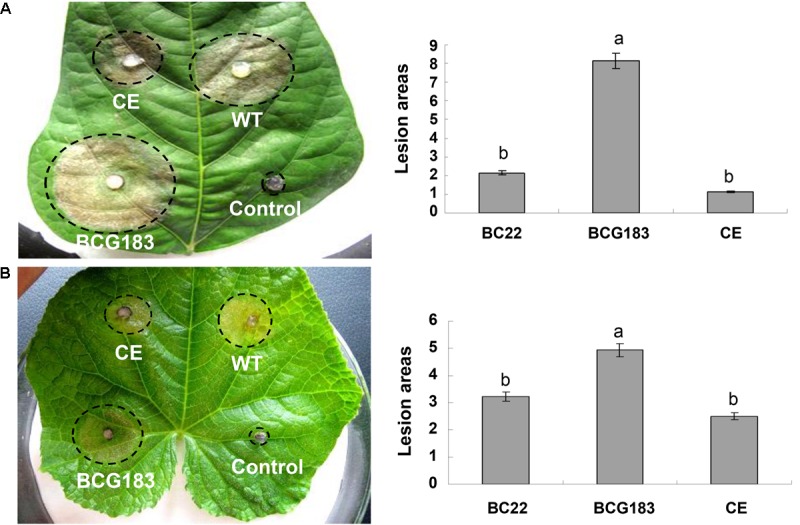
Pathogenicity analysis of BCG183 and BCG183/*BcKMO* mutants. **(A)** Pathogenicity analysis of mutants on kidney beans leaves. **(B)** Pathogenicity analysis of mutants on cucumber leaves. CE: BCG183/BcKMO mutant; a and b indicate significant differences at 0.05 probability level.

To determine the role of *BcKMO* in the pathogenicity of *B. cinerea,* culture blocks of the WT, BCG183, and BCG183*/BcKMO* strains were, respectively, inoculated onto detached bean and cucumber leaves, with blank PDA medium as the control. At 4 dpi, lesions appeared on the bean (**Figure 3A**) and cucumber (**Figure 3B**) leaves inoculated with the WT, BCG183, and BCG183/*BcKMO* strains, respectively, whereas the control did not exhibit any lesions. When compared with the WT and BCG183/*BcKMO*, the pathogenicity of BCG183 mutant on the beans (**Figure 3A**) and cucumber (**Figure 3B**) leaves dramatically increased. These results indicated that *BcKMO* is essential for the pathogenicity of *B. cinerea*.

### *BcKMO* Regulates the Activities of CWDEs, Toxins, and Acid Production

**FIGURE 4:**
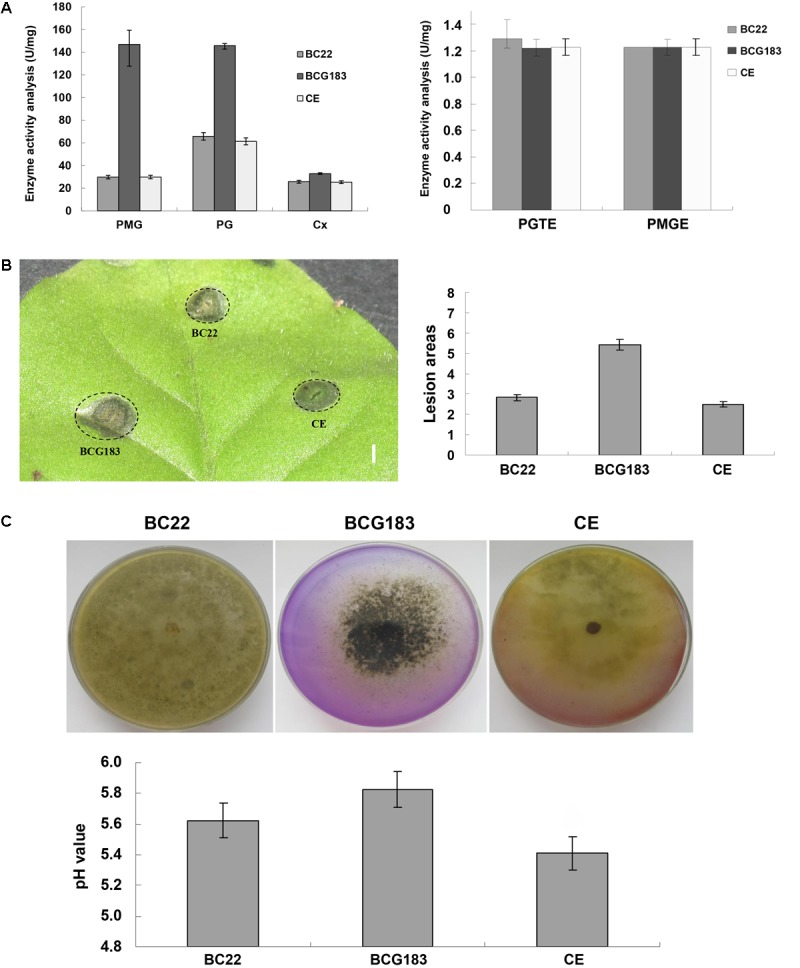
Activities of CWDEs and toxins of BCG183 and BCG183/*BcKMO* mutants. **(A)** CWDEs activity of BCG183 and BCG183/*BcKMO* mutants. **(B)** Toxins activity of BCG183 and BCG183/*BcKMO* mutants, reference bar stands for 1 cm. **(C)** Acid-producing ability of BCG183 and BCG183/*BcKMO* mutants. Acid production assays was performed on PDA medium with bromothymol blue. The pH of the PD medium inoculated with the WT, BCG183, and BCG183/*BcKMO* strains was measured.

Cell wall degrading enzymes and toxins are important for the pathogenicity of *B. cinerea*. To investigate the mechanism underlying the increased pathogenicity of the BCG183 mutant, we examined the activities of several CWDEs and toxins in the WT, BCG183, and BCG183*/BcKMO* strains. When compared with the WT and BCG183/*BcKMO,* the mutant BCG183 exhibited significantly increased activities of PMG and PG, but showed no obvious difference in the activities of CX, PGTE, and PMTE (**Figure 4A**). Furthermore, at 4 dpi, lesions appeared on tobacco leaves inoculated with crude toxins from WT, BCG183, and BCG183*/BcKMO*, and the lesion areas of BCG183 mutant were significantly larger than those of the WT and BCG183*/BcKMO* (**Figure 4B**). These findings demonstrated that *BcKMO* is involved in the activities of CWDEs and toxins in *B. cinerea*.

Interactions between *B. cinerea* and plants produce acidic substances such as oxalic acid, salicylic acid, and jasmonic acid, which alter the signaling in host cells and induce host necrosis. In the present study, the PDA medium was supplemented with bromothymol blue to measure the acid production ability of the WT, BCG183, and BCG183*/BcKMO* strains. The color of the WT and BCG183*/BcKMO* media became yellow, but that of the BCG183 medium showed no obvious change (**Figure 4C**), suggesting that the pH of the WT and BCG183*/BcKMO* media declined. To confirm the changes in pH, the WT, BCG183, and BCG183*/BcKMO* strains were cultured in PD media supplemented with bromothymol blue. The medium pH of the WT, BCG183, and BCG183*/BcKMO* at 7 dpi was 5.62, 5.83, and 5.41, respectively (**Figure 4C**), indicating that the acid production of BCG183 was weaker than that of the WT and BCG183*/BcKMO*.

### *BcKMO* Affects Hyphal Cell Walls

**FIGURE 5:**
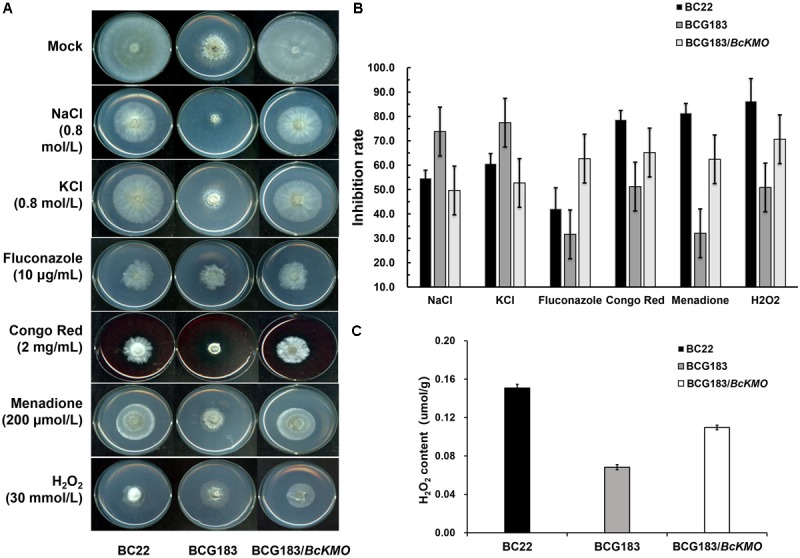
Sensitivity of BCG183 and BCG183/*BcKMO* mutants to cell wall stress. **(A)** The WT, BCG183, and BCG183*/BcKMO* strains were cultured on PDA plates with 0.8 mol/L NaCl, 0.8 mol/L KCl, 10 μg/mL fluconazole, 2 mg/mL Congo Red, 200 μmol/L menadione, and 30 mmol/L H_2_O_2_, respectively. **(B)** The inhibition rate of the three strains cultured on different media. Blank PDA was used as control. **(C)** H_2_O_2_ content of WT, BCG183, and BCG183*/BcKMO* strains.

When the WT, BCG183, and BCG183*/BcKMO* strains were inoculated onto PDA medium with 0.8 mol/L NaCl or KCl (**Figure 5A**), the BCG183 mutant exhibited remarkably higher sensitivity to NaCl and KCl, when compared with the WT and BCG183*/BcKMO* strains, and grew slowly under identical cell wall stress conditions. These results indicated that *BcKMO* deficiency reduced the resistance of *B. cinerea* to osmotic stress, suggesting a positive regulatory role of this gene in osmotic stress resistance in *B. cinerea*. A similar trend was also noted with respect to the inhibition rate (**Figure 5B**).

When the strains were inoculated onto PDA medium with 10 μg/mL fluconazole, 2 mg/mL Congo Red, 200 μmol/L menadione, and 30 mmol/L H_2_O_2_, respectively (**Figure 5A**), the sensitivity of the mutant BCG183 to fluconazole, Congo Red, menadione, and H_2_O_2_ was significantly weaker, when compared with that of the WT and BCG183*/BcKMO* strains (**Figure 5A**). These findings revealed that the cellular integrity was enhanced in the mutant BCG183, suggesting that *BcKMO* regulates the cell wall integrity of *B. cinerea*. The inhibition rate also presented a similar trend (**Figure 5B**).

The quantitative assays of H_2_O_2_ were performed in WT, BCG183, and BCG183*/BcKMO* strains. We observed that H_2_O_2_ content was declined in the mutant BCG183, comparing with WT and BCG183*/BcKMO* strains (**Figure 5C**). This result suggested that BCG183 mutant may have stronger ability to clear hydrogen peroxide.

### *BcKMO* Is Involved in cAMP and MAPK Signaling Pathways

**FIGURE 6:**
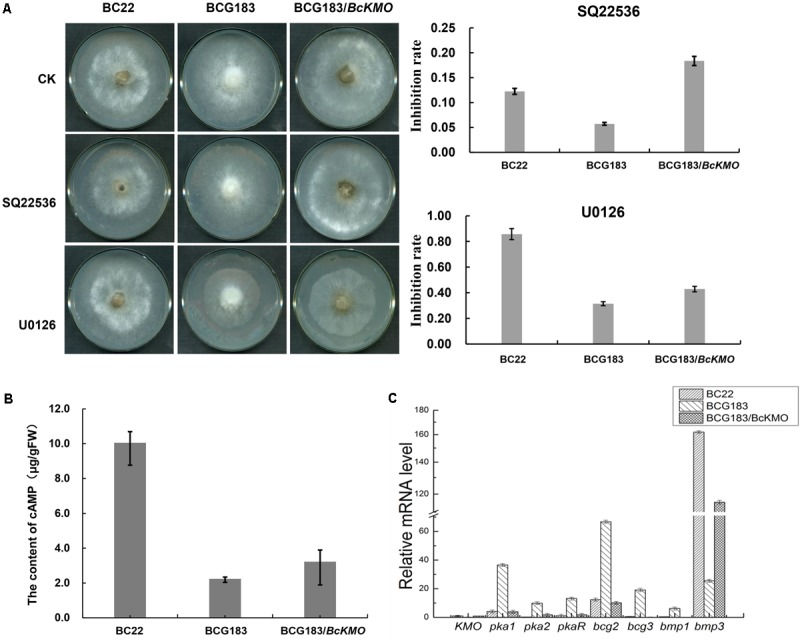
Role of *BcKMO* in the cAMP and MAPK signaling pathways. **(A)** Sensitivity of BCG183 and BCG183/*BcKMO* mutants to specific inhibitors of cAMP and MAPK signaling pathways. SQ22536, specific inhibitor of the cAMP signaling pathway; U0126, specific inhibitor of the MAPK signaling pathway. **(B)** Content of cAMP in BCG183 and BCG183/*BcKMO* mutants. **(C)** Expression levels of the key genes involved in the cAMP and MAPK signaling pathways in BCG183 and BCG183/*BcKMO* mutants.

To investigate the molecular mechanism of *BcKMO* in the regulation of the pathogenicity of *B. cinerea*, specific inhibitors of the cAMP and MAPK signaling pathways, SQ22536 and U0126, were used to detect the sensitivity of BCG183 and BCG183/*BcKMO* mutants to these inhibitors. The BCG183 mutant sensitivity to SQ22536 and U0126 was significantly weaker, when compared with that of the WT and BCG183*/BcKMO* strains (**Figure 6A**). Furthermore, the cAMP content in the mutant BCG183 was significantly lower, when compared with that in the WT and BCG183/*BcKMO* strains (**Figure 6B**). These results indicated that *BcKMO* is involved in cAMP and MAPK signaling pathways.

Quantitative real-time PCR was used to examine the expression levels of the key genes involved in cAMP and MAPK signaling pathways in BCG183 and BCG183/*BcKMO* mutants. *Pka1*, *Pka2,* and *PkaR* in the cAMP-dependent pathway; subunits of heterotrimeric GTP-binding protein encoding genes, *bcg2* and *bcg3*; and a key component gene in the MAPK signaling pathway, *bmp1*, were significantly upregulated in the mutant BCG183, whereas *bmp3*, another key gene in the MAPK signaling pathway was significantly downregulated in the BCG183 mutant (**Figure 6C**). These results demonstrated that *BcKMO* is involved in regulating the key genes involved in the cAMP and MAPK signaling pathways, namely, *pka1*, *pka2*, *pkaR*, *bcg2*, *bcg3*, *bmp1,* and *bmp3*.

**FIGURE 7:**
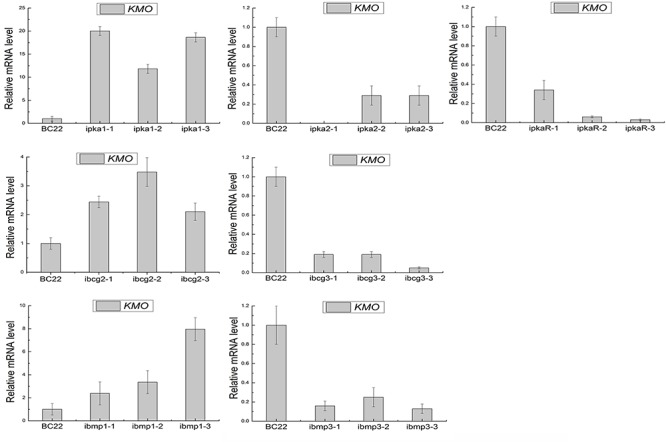
Expression levels of *BcKMO* in the RNAi mutants of the key genes involved in the cAMP and MAPK pathways.

Moreover, quantitative real-time PCR revealed that the expression levels of *BcKMO* in the RNAi mutants of *pka1*, *bcg2,* and *bmp1* were significantly higher, whereas that in the RNAi mutants of *pka2*, *pkaR*, *bcg3,* and *bmp3* were significantly lower, when compared with the *BcKMO* expression in the WT strain (**Figure 7**). These findings indicated that the key genes in the cAMP and MAPK signaling pathways, namely, *pka1*, *pka2*, *pkaR*, *bcg2*, *bcg3*, *bmp1,* and *bmp3,* regulate *BcKMO* gene expression.

## Discussion

The identification and characterization of *B. cinerea* mutants have significantly contributed to the understanding of the regulatory mechanisms involved in fungal growth, development, and pathogenicity. In this study, a novel *B. cinerea* mutant BCG183, which grew slowly, did not produce conidia and sclerotia on PDA medium, and which presented enhanced pathogenicity, was described. Using TAIL-PCR, Southern blot, RT-PCR, and bioinformatics analysis, the mutant gene was determined to be BC1G_07455 (*BcKMO*), which encodes a hypothetical protein similar to KMO with a monooxygenase FAD-binding domain and four aromatic-ring hydroxylase-like motifs. The activity of the purified BcKMO protein was noted to be 2.56 U/L, suggesting that the *BcKMO* encoded a KMO and participated in kynurenine pathway in *B. cinerea*. Subsequently, complementation experiments were designed to investigate the role of *BcKMO* in the growth, development, and pathogenicity of *B. cinerea*. The BCG183 mutant did not produce conidia and sclerotia, and presented slow growth, slender hyphae, and strong pathogenicity. Moreover, the phenotype and pathogenicity of the WT stain and *BcKMO*-complementing mutant were similar. These results demonstrated that *BcKMO* positively regulates growth and development, and negatively regulates pathogenicity of *B. cinerea*. Meanwhile, these results suggested that the kynurenine pathway might be important to growth, development, and pathogenicity in *B. cinerea*.

Kynurenine 3-monooxygenase participates in tryptophan metabolism through the kynurenine pathway. KMO belongs to the family of oxidoreductases and catalyzes the hydroxylation of L-kynurenine through the insertion of molecular oxygen into the aromatic ring of kynurenine to produce 3-hydroxy-L-kynurenine (Amaral et al., 2013). Several genes of the kynurenine pathway were discovered in several bacterial including *Bacillus cereus*, *Burkholderia fungorum*, *Ralstonia metallidurans,* and *Pseudomonas fluorescens* (Kurnasov et al., 2003a,b). Kynurenine pathway of *Pseudomonas aeruginosa*, *S. chrysomallus*, and *S. refuineus* were reported to be involved in the biosynthesis of siderophore quinolobactin, actinomycin, and benzodiazepine, respectively (Matthijs et al., 2004; Hu et al., 2007; Keller et al., 2010). KMO in yeast was directly related to the pathophysiology of Huntington disease by a mechanism that may involve reactive oxygen species (ROS) (Giorgini et al., 2005). And ROS production also can mediate or activate MAPK pathways (McCubrey et al., 2006). In this study, we found that the mutant BCG183 sensitivity to menadione and H_2_O_2_ were remarkably weak compared to the WT and BCG183/*BcKMO*. In addition, H_2_O_2_ content was declined in the mutant BCG183, comparing with WT and BCG183*/BcKMO* strains. The results indicated that the *BcKMO* gene mutation enhanced the resistance to oxidative stress, suggesting that the *BcKMO* gene negatively regulates the resistance to oxidative stress, and then influences the MAPK signaling pathways in *B. cinerea*.

The conidia of *B. cinerea* play an important role in disease cycle and propagation. Therefore, it is generally believed that inhibition of formation and development of *B. cinerea* conidia could alleviate or effectively control the occurrence of gray mold. However, the relationship between the conidiation and pathogenicity of *B. cinerea* is not yet clear. In a previous study, a Δ*bcpka1* mutant was noted to sporulate in plants, and although its growth rate, conidiation, and conidial germination were not impaired, the mutant failed to produce soft rots of leaves (Schumacher et al., 2008b). Furthermore, deletion of genes encoding the MAPK, *Bmp1* and *BcSAK1*, resulted in non-pathogenic mutants. It has been reported that the Δ*bmp1* mutant produced normal conidia and exhibited decreased growth rates (Zheng et al., 2000), whereas the Δ*bcsak1* mutant did not produce conidia and showed decreased growth rates (Gronover et al., 2001). Moreover, deletion of the MAP kinase gene, *BMP3*, has been found to result in reduced conidiation, growth rates, and virulence (Rui and Hahn, 2007). In the present study, the BCG183 mutant exhibited decreased growth rates and did not produce conidia, but presented obviously enhanced pathogenicity. These results suggest that conidiation and pathogenicity of *B. cinerea* are not necessarily correlated.

The major pathogenic mechanism of *B. cinerea* involves multiple CWDEs and pathogenic toxins (Juge, 2006; Choquer et al., 2007). Therefore, in the present study, the role of *BcKMO* in regulating CWDEs and pathogenic toxins activities was investigated. The activities of PMG, PG, and toxins in the BCG183 mutant were significantly higher, when compared with those in the WT and BCG183/*BcKMO* strains. In addition, inactivation of *BcKMO* upregulated the expression levels of *Bcp1*, *god1*, *Bcpg1*, and *Sod1* (data not shown). These results suggest that *BcKMO* is involved in regulating CWDEs and toxins activities.

Oxalic acid is known to be an important factor in the pathogenesis of *S. sclerotiorum* (Walz et al., 2008), and favors plant cell wall degradation by decreasing the cellular pH close to the optimum for CWDE and removes Ca^2+^ ions bound to pectins. In addition, OA inhibits the plant oxidative burst and defense mechanisms and reduces plant programmed death (PCD), thereby increasing the pathogenicity of the pathogen. However, PCD has a clear role in promoting pathogen growth in some host–pathogen interactions (Greenberg and Yao, 2004). Studies on *B. cinerea* in tobacco and *Arabidopsis* suggested that the fungus may even need the hypersensitive reaction to achieve full pathogenicity (Govrin and Levine, 2000; Dickman et al., 2001). Indeed, *Arabidopsis* mutants with a delayed or reduced cell death response were generally more resistant to *B. cinerea* infection, whereas mutants in which cell death was accelerated were more susceptible (Van Baarlen et al., 2007). Depending on the timing, strength, and host, PCD has an important role in *B. cinerea* virulence. In the present study, the pathogenicity of the mutant on kidney beans and cucumber leaves was dramatically increased, whereas acid production of the BCG183 mutant significantly decreased, suggesting that the weak acid level in the mutant might have promoted oxidative burst and PCD resulting in increased pathogenicity. In contrast, the low pH in the WT and BCG183/*BcKMO* strains might have inhibited oxidative burst and PCD, thus limiting their pathogenicity.

Some signal transduction components of *B. cinerea*, especially those involved in the MAPK and cAMP-dependent pathways, are known to regulate the fungal development and pathogenicity. In addition, studies have demonstrated that ROS can induce or mediate the activation of the MAPK pathways (McCubrey et al., 2006). Therefore, in the present study, the mutant BCG183 sensitivity to menadione and H_2_O_2_ were remarkably weak compared to the WT and BCG183/BcKMO. The *BcKMO*-mediated pathway was further investigated by detecting the sensitivity of BCG183 and BCG183/*BcKMO* mutants to specific inhibitors of cAMP and MAPK signaling pathways, as well as by determining the cAMP content in these mutants. The sensitivity of the BCG183 mutant to signaling pathway specific inhibitors, SQ22536 and U0126, was remarkably weak (**Figure 6A**), and the content of cAMP was significantly reduced (**Figure 6B**). Thus, it can be concluded that *BcKMO* is involved in cAMP and MAPK signaling pathways. To further analyze the relationship between *BcKMO* and the key genes involved in the cAMP and MAPK signaling pathways, the expression levels of these genes in the BCG183 and BCG183/*BcKMO* mutants as well as the expression of *BcKMO* in the RNAi mutants of these genes were examined by quantitative real-time PCR. While *pka1*, *pka2*, *pkaR*, *bcg2*, *bcg3*, and *bmp1* were significantly upregulated, *bmp3* was significantly downregulated in the BCG183 mutant (**Figure 6C**), indicating that *BcKMO* negatively regulates *pka1*, *pka2*, *pkaR*, *bcg2*, *bcg3*, and *bmp1* and positively regulates *bmp3*. The expression of *BcKMO* was obviously upregulated in the RNAi mutants of *pka1*, *bcg2,* and *bmp1*, but significantly downregulated in the RNAi mutants of *pka2*, *pkaR*, *bcg3,* and *bmp3* (**Figure 7**), suggesting that *BcKMO* is negatively regulated by *pka1*, *bcg2*, and *bmp1* and positively regulated by *pka2*, *pkaR*, *bcg2,* and *bmp3*. These findings provide a foundation for further examination of the molecular mechanisms of *BcKMO* in regulating the growth, development, and pathogenicity of *B. cinerea*.

## Conclusion

The *BcKMO* gene, which encodes a KMO, was found to positively regulate growth and development, but to negatively control pathogenicity of *B. cinerea*. This gene was noted to be involved in the regulation of CWDEs activity, toxins activity, acid production, and cell wall integrity, and participate in cAMP and MAPK signaling pathways of *B. cinerea*.

## Author Contributions

JX and DG conceived and designed the experiments. XY, JZ, and MW performed the experiments. KZ, XY, JZ, MW, FZ, PL, and HC analyzed the experimental data. KZ, XY, JH, and JX contributed reagents/materials/analysis tools. KZ, JX, and JD wrote the paper. All authors read and approved the final manuscript.

## Conflict of Interest Statement

The authors declare that the research was conducted in the absence of any commercial or financial relationships that could be construed as a potential conflict of interest.

## Footnotes

1 http://www.broad.mit.edu/annotation/genome/botrytis_cinerea

2 https://blast.ncbi.nlm.nih.gov/Blast.cgi

## Supplementary Material

FIGURE S1 Identification of T-DNA insertion site in the BCG183 mutant. **(A)** PCR identification of the BCG183mutant with the specific primers of hph gene. M: DNA marker; 1, 3: Mutant BCG183; 2: WT. A 800-bp fragment was amplified from the BCG183 mutant DNA, but not from the WT genomic DNA. **(B)** Southern hybridization analysis of T-DNA insertion in the BCG183 mutant. M: DNA marker; 1: Mutant BCG183. The specific fragment of hph gene was used as the DNA probe of Southern hybridization. A single band was detected by Southern blot of the BCG183 genomic DNA, suggesting a single-copy T-DNA insertion in the BCG183 genomic DNA. **(C)** Electrophoresis of TAIL-PCR products. M: DNA marker; 1, 2: BCG183 mutant. The flanking sequence of the T-DNA insertion site was amplified by TAIL-PCR from the BCG183 genomic DNA. **(D)** Illustration of the BC1G_07455 locus with the T-DNA insertion in the BCG183mutant. Alignment of the sequence of the TAIL-PCR product with *Botrytis cinerea* gene database revealed insertion in the 3′-end of the coding region of BC1G_07455. **(E)** Expression level of BC1G_07455 detected by semi-quantitative RT-PCR. The expression of BC1G_07455 in the BCG183 mutant was significantly lower than that in the WT strain.

FIGURE S2 Identification of T-DNA insertion site in BCG183 mutant. **(A)** Construction of prokaryotic expression vector of *BcKMO*. M: DNA marker; 1: Identification of pGEX4T-1-BcKMO-GST by restriction enzyme digestion. **(B)** Relationship between different times of induction and expression levels of BcKMO-GST. M: Protein Marker; 1: Without IPTG; 2–6: Expression of BcKMO-GST with IPTG induction at 4, 8, 12, 16, and 20 h. **(C)** Purification of BcKMO-GST protein. M: Protein Marker; 1: Purified BcKMO-GST protein; 2: Supernatant before purification. **(D)** Western blot analysis of BcKMO-GST protein. M. Protein Marker; 1: Supernatant before purification; 2: Purified BcKMO-GST protein. **(E)** Standard curve of KMO activity measurement using the Human KMO ELISA Kit.

FIGURE S3 Complementation of *BcKMO*. **(A)** Vector construction of pBARKS1-*BcKMO*-*GFP*. **(B)** Enzyme digestion identification of pBARKS1-*BcKMO*-eGFP; M: DNA marker. **(C)** PCR identification of the transformant (M: DNA marker; 1, 2: PCR amplification of the WT and transformant with *bar*-specific primers; 3, 4: PCR amplification of the WT and transformant with *GFP*-specific primers). **(D)** Southern blot identification of the transformant (M: DNA marker; 1: WT; 2, 3: transformant). **(E)** Real-time PCR identification of the transformant. BC22: WT; CE: BCG183*/BcKMO* mutant. Quantitative real-time PCR analysis of *BcKMO* expression was performed with *BcKMO*-specific primers. Transcript levels of *BcKMO* in the transformant were significantly higher than those in the WT strain.

TABLE S1 Primers used in this study.
