# Supplementary figures and images for: The Kynurenine 3-Monooxygenase Encoding Gene, BcKMO, Is Involved in the Growth, Development, and Pathogenicity of Botrytis cinerea
Source: Front Microbiol. 2018 May 18;9:1039. doi: 10.3389/fmicb.2018.01039 (PMC5968091; doi:10.3389/fmicb.2018.01039)

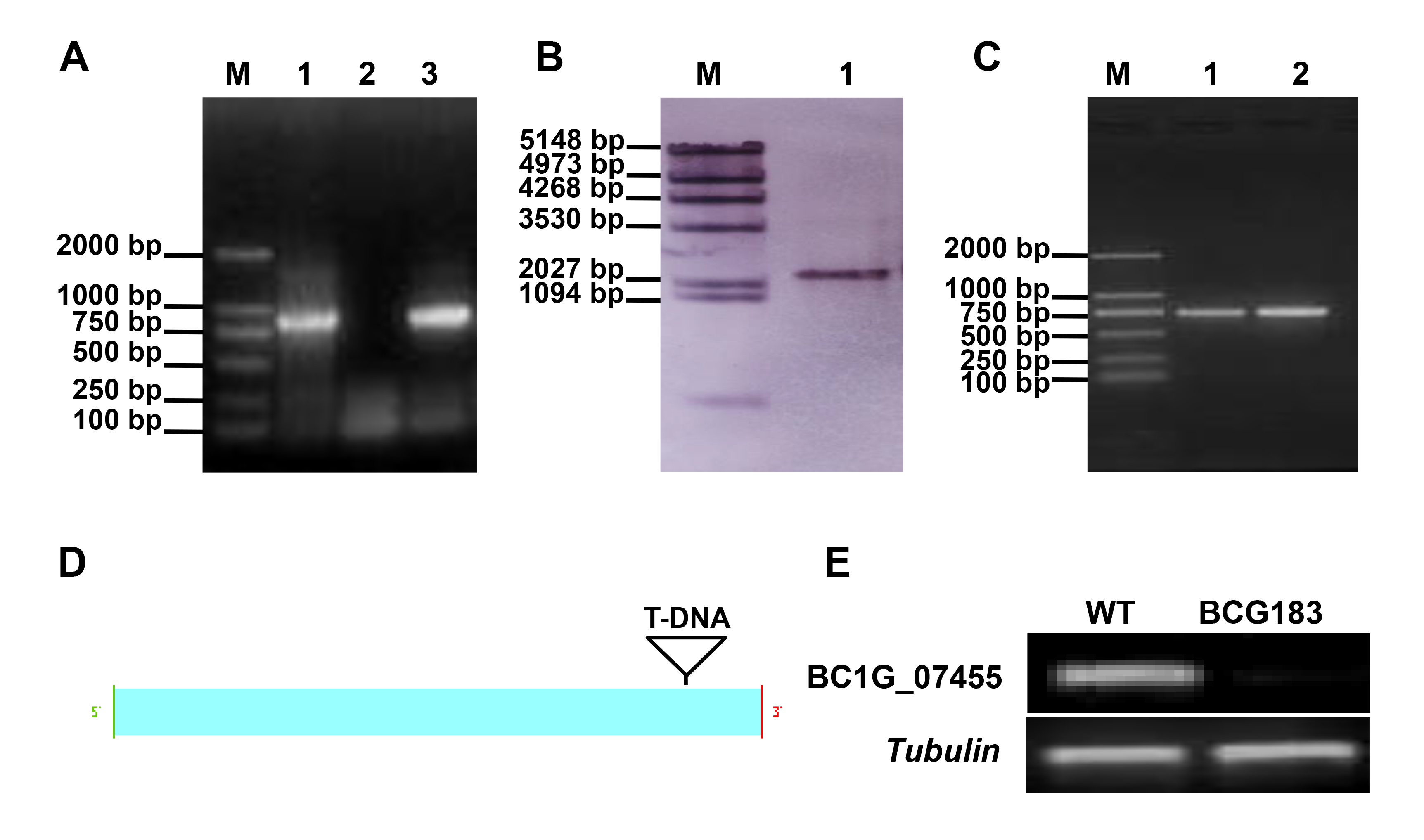

Supplement: FIGURE S1 — Identification of T-DNA insertion site in the BCG183 mutant. (A) PCR identification of the BCG183mutant with the specific primers of hph gene. M: DNA marker; 1, 3: Mutant BCG183; 2: WT. A 800-bp fragment was amplified from the BCG183 mutant DNA, but not from the WT genomic DNA. (B) Southern hybridization analysis of T-DNA insertion in the BCG183 mutant. M: DNA marker; 1: Mutant BCG183. The specific fragment of hph gene was used as the DNA probe of Southern hybridization. A single band was detected by Southern blot of the BCG183 genomic DNA, suggesting a single-copy T-DNA insertion in the BCG183 genomic DNA. (C) Electrophoresis of TAIL-PCR products. M: DNA marker; 1, 2: BCG183 mutant. The flanking sequence of the T-DNA insertion site was amplified by TAIL-PCR from the BCG183 genomic DNA. (D) Illustration of the BC1G_07455 locus with the T-DNA insertion in the BCG183mutant. Alignment of the sequence of the TAIL-PCR product with Botrytis cinerea gene database revealed insertion in the 3′-end of the coding region of BC1G_07455. (E) Expression level of BC1G_07455 detected by semi-quantitative RT-PCR. The expression of BC1G_07455 in the BCG183 mutant was significantly lower than that in the WT strain. [file Image_1.JPEG]

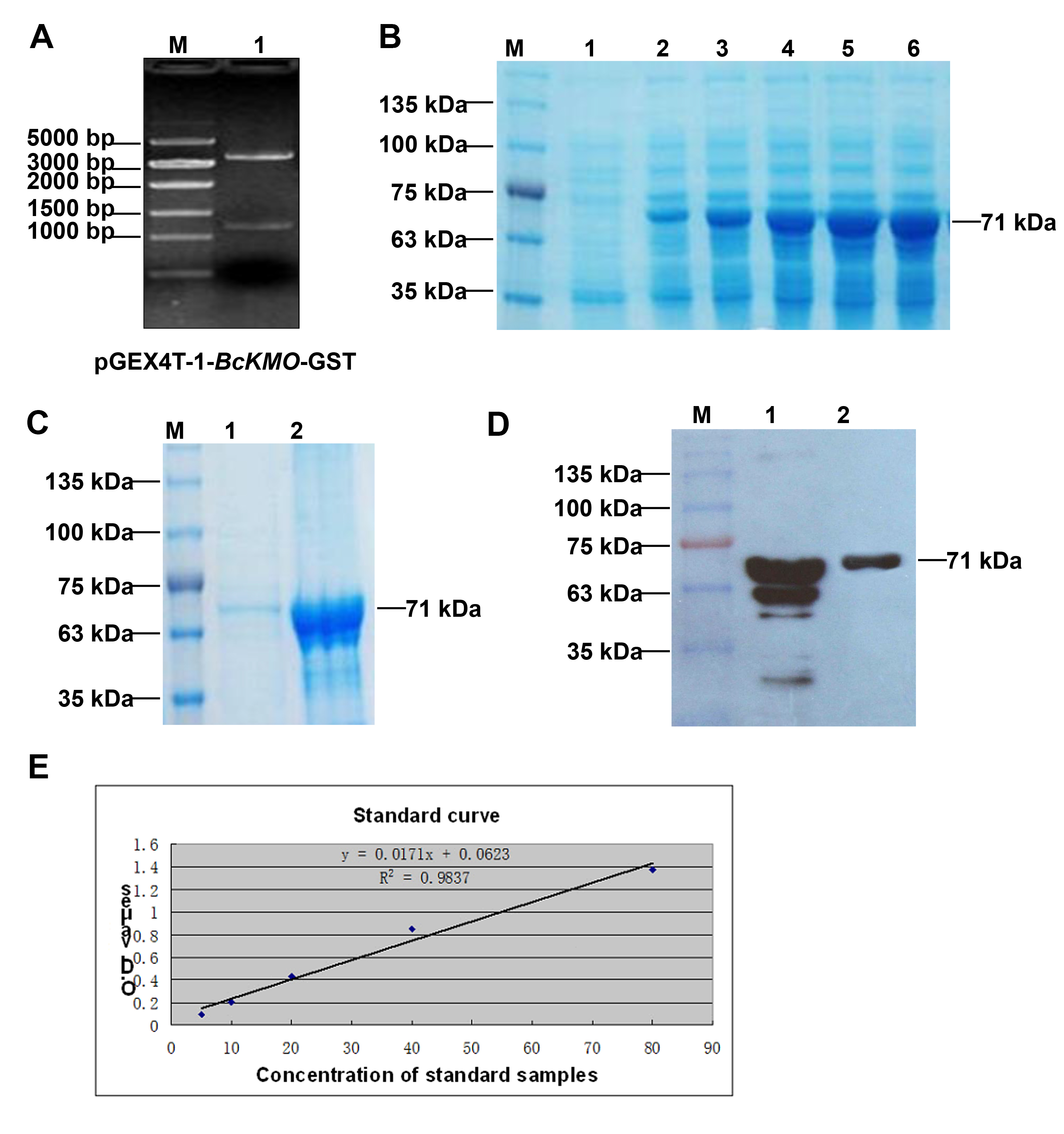

Supplement: FIGURE S2 — Identification of T-DNA insertion site in BCG183 mutant. (A) Construction of prokaryotic expression vector of BcKMO. M: DNA marker; 1: Identification of pGEX4T-1-BcKMO-GST by restriction enzyme digestion. (B) Relationship between different times of induction and expression levels of BcKMO-GST. M: Protein Marker; 1: Without IPTG; 2–6: Expression of BcKMO-GST with IPTG induction at 4, 8, 12, 16, and 20 h. (C) Purification of BcKMO-GST protein. M: Protein Marker; 1: Purified BcKMO-GST protein; 2: Supernatant before purification. (D) Western blot analysis of BcKMO-GST protein. M. Protein Marker; 1: Supernatant before purification; 2: Purified BcKMO-GST protein. (E) Standard curve of KMO activity measurement using the Human KMO ELISA Kit. [file Image_2.JPEG]

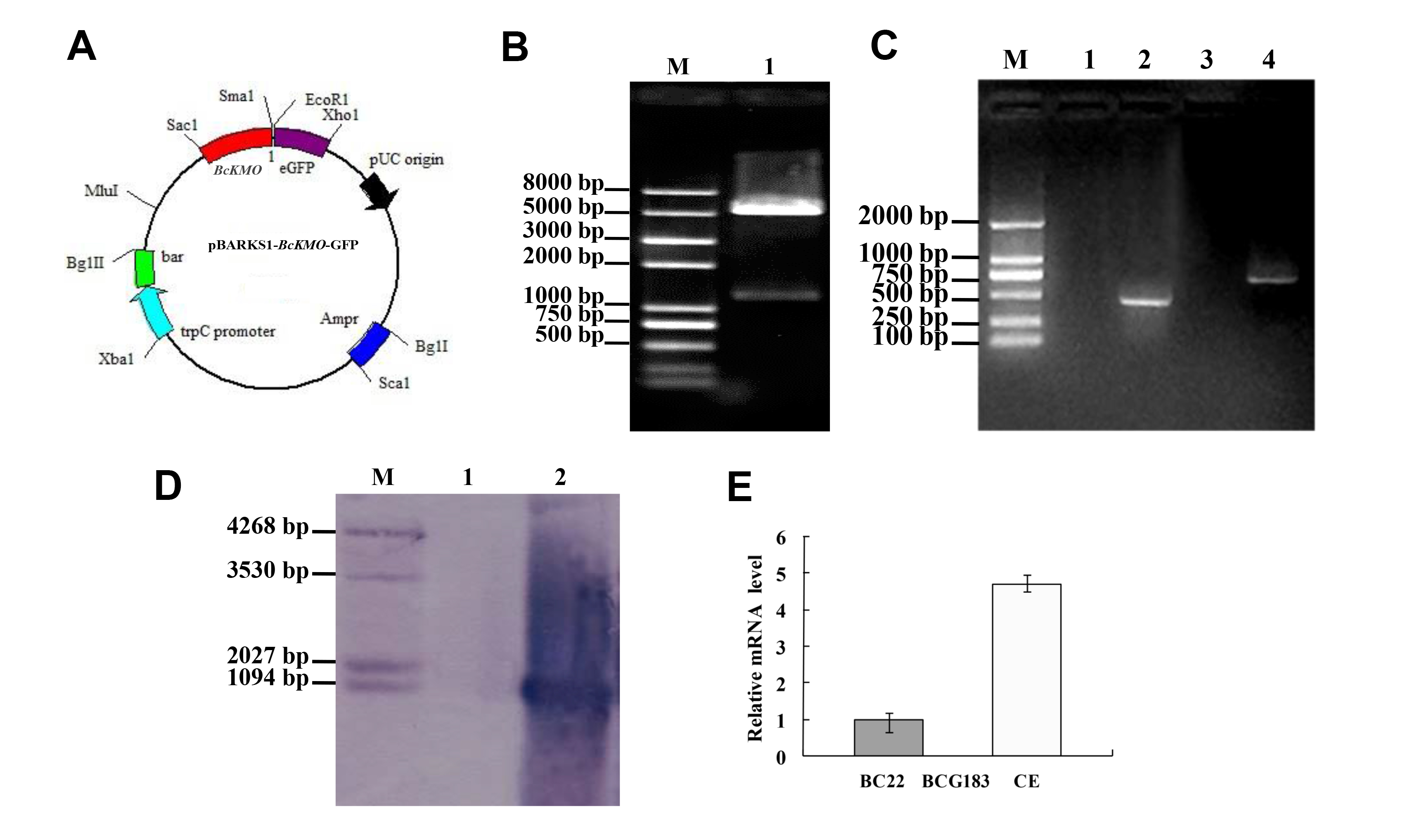

Supplement: FIGURE S3 — Complementation of BcKMO. (A) Vector construction of pBARKS1-BcKMO-GFP. (B) Enzyme digestion identification of pBARKS1-BcKMO-eGFP; M: DNA marker. (C) PCR identification of the transformant (M: DNA marker; 1, 2: PCR amplification of the WT and transformant with bar-specific primers; 3, 4: PCR amplification of the WT and transformant with GFP-specific primers). (D) Southern blot identification of the transformant (M: DNA marker; 1: WT; 2, 3: transformant). (E) Real-time PCR identification of the transformant. BC22: WT; CE: BCG183/BcKMO mutant. Quantitative real-time PCR analysis of BcKMO expression was performed with BcKMO-specific primers. Transcript levels of BcKMO in the transformant were significantly higher than those in the WT strain. [file Image_3.JPEG]
