# Supplementary material for: The Kynurenine 3-Monooxygenase Encoding Gene, BcKMO, Is Involved in the Growth, Development, and Pathogenicity of Botrytis cinerea
Source: Front Microbiol. 2018 May 18;9:1039. doi: 10.3389/fmicb.2018.01039 (PMC5968091; doi:10.3389/fmicb.2018.01039)
Supplement: TABLE S1 — Primers used in this study. [file Table_1.DOC]

**Table S1.** **Primers used in this study.**

| Purpose | Gene name | Primer name | Sequence (5’ - 3’) |
| --- | --- | --- | --- |
| Identification of mutant BCG183 | *hph* | hph-F | CGACAGCGTCTCCGACCTGA |
|  |  | hph-R | CGCCCAAGCTGCATCATCGA |
| TAIL-PCR | T-DNA | LB1 | GGGTTCCTATAGGGTTTCGCTCATG |
|  |  | LB2 | CATGTGTTGAGCATATAAGAAACCCT |
|  |  | LB3 | GAATTAATTCGGCGTTAATTCAGT |
|  |  | AD4 | TCGTNCGNACNTAGGA |
| Semi-quantitative RT-PCR | *Tubulin* | Tublin-F | TCTCCGTAAGATGGGTTG |
|  |  | Tublin-R | ACTGGGCTAAGGGTCATT |
|  | BC1G_07455 | BC1G_07455-F | GTTCGTCGCTATCTCTATCC |
|  |  | BC1G_07455-R | ATCTCCCTTGTCCGACTTTG |
| Construction of *BcKMO* complementing vector | *BcKMO* | KMO-F | ACTGGTACTGCGCCTCTTCTC |
|  |  | KMO-R | ATCTCCCTTGTCCGACTTTG |
| PCR analysis of transformant | GFP | GFP-F | ATGAGTAAAGGAGAAGAACTTTTCA |
|  |  | GFP-R | TTACCACCACCACCAAGACC |
|  | *bar* | bar-F | CTGCTCGACGCTACTGC |
|  |  | bar-R | GCACAACCATGCAGACCTTT |
| Quantitative real-time PCR | *Tubulin* | Tubulin-F | ACATGCTCTGCCATTTTCCG |
|  |  | Tubulin-R | TTGTTAGGGATCCACTCAACGAAG |
|  | *BcKMO* | BcKMO-F | TGAGAATCGACGTGTGGTTATT |
|  |  | BcKMO-R | GTATCTCCCTTGTCCGACTTTG |
|  | *Pka1* | pka1-F | TCAGAAGAGGACGATGAGGATG |
|  |  | pka1-R | TTGCCACGAAGTTGAAACCA |
|  | *Pka2* | pka2-F | GGCCAAATTCTATGCTGCT |
|  |  | pka2-R | CATCTGGGTGAATGTAGGGA |
|  | *PkaR* | PkaR-F | CCGTATCACCAGGAACAGCA |
|  |  | PkaR-R | ATGTCCTAACCCATTTCCGTC |
|  | *bcg2* | bcg2-F | AAGTTTGGTTTCTCCGATTTCC |
|  |  | bcg2-R | CGGTATCGGTGGCGTTTG |
|  | *bcg3* | bcg3-F | ATCCAGCGAACAAGGAATACG |
|  |  | bcg3-R | GGTGCCGAATCCATCAAATAG |
|  | *bmp1* | bmp1-F | CTATCAAACCCTGCGAGCCT |
|  |  | bmp1-R | CTGGTCGCAACATATTCTGTCA |
|  | *bmp3* | bmp3-F | ACAACATGTCCGTGTTCAACCTG |
|  |  | bmp3-R | CACGCCAGCTTGCTTGTTCT |
